# Long-Term Outcomes After Childhood Stroke

**DOI:** 10.3390/pediatric18020050

**Published:** 2026-04-01

**Authors:** Kerttu Kivisikk, Pilvi Ilves, Mairi Männamaa, Eve Õiglane-Shlik, Nigul Ilves, Norman Ilves, Inga Talvik, Dagmar Loorits, Pille Kool, Rael Laugesaar

**Affiliations:** 1 Department of Pediatrics, Institute of Clinical Medicine, University of Tartu, 50406 Tartu, Estoniarael.laugesaar@kliinikum.ee (R.L.); 2 Children’s Clinic, Tartu University Hospital, 50406 Tartu, Estonia; 3 Department of Radiology, Institute of Clinical Medicine, University of Tartu, 50406 Tartu, Estonia; pilvi.ilves@kliinikum.ee (P.I.); pille.kool@kliinikum.ee (P.K.); 4 Radiology Clinic, Tartu University Hospital, 50406 Tartu, Estonia; 5 Tallinn Children’s Hospital, 13419 Tallinn, Estonia

**Keywords:** arterial ischemic stroke, cerebral venous sinus thrombosis, childhood stroke, hemorrhagic stroke, outcome

## Abstract

The aim of this study was to assess long-term outcomes in patients with different vascular types of childhood stroke. Methods: Data for children with childhood stroke (aged 29 days to 18 years) were collected from the Estonian Pediatric Stroke Database. Outcomes (death, recurrent stroke, epilepsy, neurodevelopmental outcome by pediatric stroke outcome measure (PSOM)) were assessed at a minimum of two years after stroke. Results: Long-term outcome data were available for 44 patients with childhood stroke (including three patients who died of stroke). According to the PSOM, based on gender, age, location of stroke and epilepsy, there were no differences in outcomes, but patients with a Pediatric NIH Stroke Scale (PedNIHSS) score of ≥6 had worse outcomes compared to patients with a score of <6. Children with arterial hemorrhagic stroke (AHS) were more likely to die, suffer from epilepsy and develop problems in the cognition/behavior PSOM subscale compared to children with arterial ischemic stroke (AIS). Combined poor outcomes (epilepsy, PSOM ≥ 1, recurrent stroke, mortality) occurred in 75% (33/44) of all patients with long-term outcome data. Conclusions: Combined poor outcomes occurred in 75% of the patients with childhood stroke. Patients with AHS showed higher mortality and worse long-term outcomes compared to patients with AIS in certain neurodevelopmental domains.

## 1. Introduction

Childhood stroke is a cerebrovascular event which occurs between 28 days and 18 years of age [1] and has emerged as an important cause of neurological disability in children. The incidence of childhood stroke is 1.3 to 17.96 per 100,000 person-years worldwide [2,3] and 2.73 per 100,000 person-years in Estonia [4].

According to its vascular distribution and mechanism of development, there are three main subtypes of childhood stroke: (1) arterial ischemic stroke (AIS), (2) arterial hemorrhagic stroke (AHS), and (3) cerebral venous sinus thrombosis (CVST) [5,6].

AIS forms the biggest subgroup of childhood stroke with 51–59% and is estimated to affect 1.0 to 2.0 per 100,000 children (non-neonates) annually [7,8,9]. AHS makes up 32–41% of childhood stroke, with an incidence of ≈1 to 1.7 per 100,000 per year [7,10]. CVST accounts for 9% of childhood stroke with an incidence of 0.25 cases per 100,000 children per year [4].

The signs and symptoms of acute stroke in children are similar to those in adults: the main symptoms of acute stroke are hemiparesis and hemifacial weakness (67–90%), speech and language disturbance (20–50%), and vision disturbance (5–15%). The main non-localized symptoms are headache (20–50%) and altered mental status (17–38%) [11]. Seizures at stroke onset affect 20% to 36% of patients [9,12].

Stroke in childhood is a cause of long-term disability and increased mortality. Hemiparesis has been described as long-term disability in 56% of the patients with AIS [13] and in 35% of the patients with AHS [14]. However, the issues that these children face are not only sensorimotor, but also cognitive and academic [15,16]. At least one residual complaint is present in 62% of children with ischemic stroke [6]. Special education is needed for 39.5% of children with childhood stroke [6], and 55.6% of AIS patients with cortical lesions have psychological disorders (attention-deficit hyperactivity disorder, intellectual disability, learning disability, depression, anxiety, language disorder, and autism spectrum disorder) [17]. Post-stroke epilepsy is described in 10–41% of children with AIS, and the presence of epilepsy has been correlated with worse outcomes one year after stroke [18,19].

There are not many factors that have been associated with long-term outcomes after childhood stroke, but it has been shown that in patients with childhood AIS, Pediatric NIH Stroke Scale score predicts global deficits in the pediatric stroke outcome measure (PSOM) at 3 and 12 months after childhood stroke [20].

The data of global long-term outcome in children with childhood stroke with different vascular etiologies is scarce. Most studies on the outcomes of childhood stroke have focused on one specific problem (language, cognitive, or motor outcomes, etc.) [19,21] or on one specific vascular type of stroke, most often AIS [19,21,22,23,24]. A few earlier studies describe long-term outcomes across all stroke subtypes [25] with different aspects of the development.

The aim of this study was to assess long-term global outcomes in patients with different vascular types of childhood stroke and to identify predictors of poor neurodevelopmental outcomes.

## 2. Methods

### 2.1. Study Design

For this population-based prospective pilot study, patients were identified from the Estonian Pediatric Stroke Database, which contains the data of children with childhood stroke collected within an epidemiological study (1995–2006) [4] and prospectively thereafter. Almost all children with stroke were diagnosed at two tertiary children’s clinics in the country (Tallinn Children’s Hospital and Children’s Clinic of Tartu University Hospital), which facilitates conduct of epidemiological studies. To find any patients missing from the database, additional health record searches applying the International Classification of Diseases-10 [26] codes (I60-I69, G81, G83.1, G83.2, G83.3 and G83.9) were made at these two tertiary children’s hospitals (2007–2022). Not to miss any adolescent cases, a similar search was made at the Neurology Clinic of Tartu University Hospital and at the North Estonia Medical Center.

All radiological images of the patients with childhood stroke in the Estonian Pediatric Stroke database were independently reviewed from the population-based Estonian Picture Archiving System by three neuroradiologists (P.I., D.L, No.I.) who were blinded to the clinical outcome of the patients and the diagnosis of stroke. The type of the vascular genesis of childhood stroke was based on the consensus agreement of the three neuroradiologists.

AIS was diagnosed as an acute focal neurological deficit caused by a sudden loss of blood circulation in the cerebral artery due to thrombosis or emboli [27]. AHS was diagnosed as a spontaneous intraparenchymal or nontraumatic subarachnoid hemorrhage [28]. CVST was defined as a symptomatic thrombosis of cerebral sinuses or veins accompanied or not by cerebral (hemorrhagic) infarction [29]. In all cases, acute stroke was confirmed by computed tomography (CT) and/or magnetic resonance imaging (MRI) and diagnosis of stroke was based on imaging analysis and clinical correlation.

The etiology of stroke was specified from physical medical records and, starting from 2006, from digital medical records of children. Risk factors were divided into six categories according to a modified version of Mackay and Steinlin 2019 [30]: (1) intracranial vasculopathy (focal cerebral arteriopathy and cerebral vasculitis for AIS and arteriovenous malformation (AVM) and aneurysm for AHS); (2) hematological (acquired or inherited thrombophilia for AIS and CVST, and hemophilia, thrombocytopenia or vitamin K deficiency for AHS); (3) cardiac risk factors (congenital heart disease, acquired heart disease or cardiac surgery); (4) infections (systemic or local head and neck infections); (5) trauma; and (6) idiopathic. For all patients, initial Pediatric NIH Stroke Scale score was available.

### 2.2. Participants

All patients with childhood stroke in the Pediatric Stroke Database were reviewed by a child neurologist and a neuroradiologist before inclusion in the outcome study. Patients for the follow-up study had to fulfill all the inclusion criteria: (1) stroke type AIS, AHS or CVST confirmed by CT and/or MRI; (2) age at diagnosis of stroke between 29 days of life and 18 years of age; (3) time of at least two years after stroke; and (4) age at the time of participation in the study up to 18 years 11 months. The exclusion criteria were (1) concomitant disease of the central nervous system (cerebral anoxia, brain anomalies) and (2) genetic or other diseases which are unrelated to stroke but cause developmental delay.

All eligible patients/caregivers of the patients in the Pediatric Stroke Database were contacted by phone and the patients who gave consent to participate in the outcome study were evaluated between January 2009 and November 2023. All patients identified with childhood stroke in the Pediatric Stroke Database were assessed for mortality and epilepsy until 1 December 2023. Written informed consent for participation in the study was obtained from the childrens’ parents and from the children aged seven years or older.

The study was approved by the Research Ethics Committee of the University of Tartu (decisions 170/T-6 from 28 April 2008; 233/T-10 from 2 January 2014; 302/M-20 from 16 March 2020; 364/M-15 from 16 May 2022).

### 2.3. Outcome Assessments

The patients were assessed by a multidisciplinary team of specialists including a pediatric neurologist, an occupational therapist, a speech therapist, a clinical psychologist, a physiotherapist, and a radiologist. Medical history, including initial PedNIHSS score, was obtained from past medical records and additional information was obtained from the parents and the children. All assessments were performed in an outpatient clinic within one day or within one hospitalization, if needed.

The main outcome measure used was the pediatric stroke outcome measure (PSOM) [31], a disease-specific measure of neurological recovery after stroke in children. The PSOM is composed of 5 subscales (right and left sensorimotor, language production, language comprehension, and cognitive/behavior), which rate neurologic impairment ranging from 0 to 2 (0 = none, 0.5 = mild, 1 = moderate, 2 = severe). When scoring the PSOM subscales, we incorporated not only bedside testing by a pediatric neurologist, but also assessments by different medical specialists. The subscales were added to generate a score ranging from 0 (no deficit) to 10 (severe deficit). Normal outcome was defined as a score of 0 in all five subscales, mild deficit as a score of 0.5 in one subscale only, moderate deficit as an overall score of 1–1.5, and severe deficit as an overall score of 2–10. Outcome was dichotomized as good (total PSOM score < 1) or poor (total PSOM score ≥ 1) [32].

Epilepsy was diagnosed by a pediatric neurologist at a third-level children’s hospital after the acute stage of stroke as follows: at least two unprovoked seizures occurring >24 h apart or one unprovoked seizure with high recurrence risk, or diagnosis of epilepsy syndrome [33]. The prevalence of epilepsy was based on the all-Estonian digital medical records and on the all-Estonian database of digital prescriptions. For assessing combined outcomes in all patients with known outcomes, we combined the PSOM-based poor outcome, epilepsy, stroke mortality, and recurrent stroke. While these factors vary considerably in clinical severity, they all contribute significantly to the burden of stroke on patients and families.

A follow-up MRI was performed for this study without anesthesia at the age of 8–18 years (*n* = 22), with a 3 T Philips Achieva scanner using an 8 channel SENSE head coil (Philips Medical Systems, Best, The Netherlands). For children aged <8 years or for children who declined to undergo a new scan (*n* = 17), a previous follow-up MRI done at least one month after stroke was used for analyzing the size and location of the brain lesion of stroke. Two patients did not undergo the follow-up MRI.

The lesions of stroke were evaluated as right- or left-sided or those with bilateral involvement. The location of damage was evaluated as (1) cortical or (2) noncortical involving the periventricular white matter, basal ganglia, brain stem, or cerebellum.

### 2.4. Statistical Analysis

Statistical evaluation was performed using the statistical package SAS version 9.4 (SAS Institute, Cary, NC, USA). Prior to further analysis, the normality of the data was evaluated using the Shapiro–Wilk test. For the description of the proportions of the patient characteristics, medians with the interquartile range (IQR) were reported. Difference between the groups was analyzed by the nonparametric Mann–Whitney *U* test and the nonparametric Kruskal–Wallis test for continuous variables. To compare the proportions, the chi-square test and Fisher’s Exact test (when expected values were <5) were used. The odds ratio (OR) with the 95% confidence interval (CI) was estimated as the measure of association. All analyses were carried out using a significance level of 5%.

## 3. Results

### 3.1. Participants and Study Group

**Figure 1:**
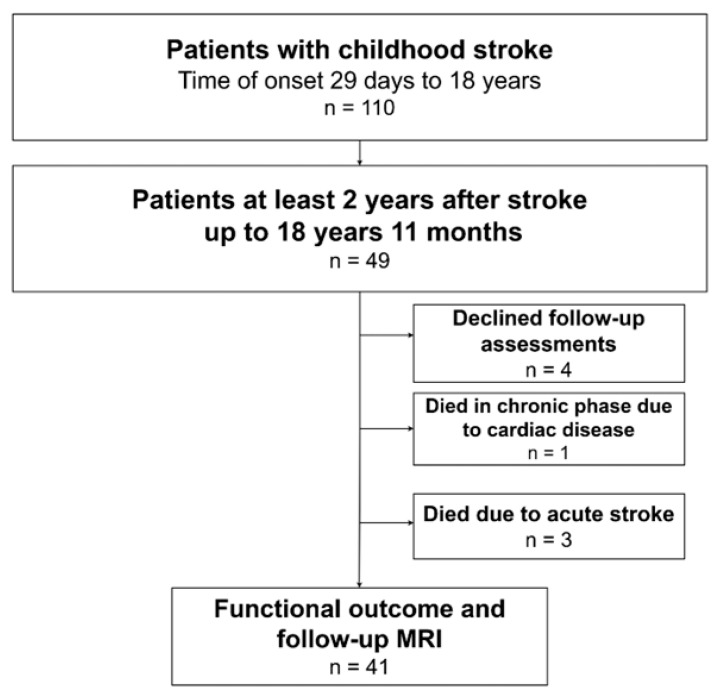
Patient recruitment in the neurocognitive outcome study. MRI, magnetic resonance imaging.

A total of 110 patients with childhood stroke were identified from the Estonian Pediatric Stroke Database, of these, 49 were aged up to 18 years 11 months, with stroke diagnosed at least 2 years earlier. A flow chart of the patients is presented in Figure 1. The 49 patients were divided into three subgroups according to the vascular type of stroke: (1) AIS (*n* = 28, 57%), (2) AHS (*n* = 16, 33%) and (3) CVST (*n* = 5, 10%). The occurrence of childhood stroke revealed male predominance (55%) without differences between the different vascular subgroups (Table 1). Stroke was diagnosed at a median age of 4.0 years (IQR 1.1–8.6), with no differences between the vascular subgroups of stroke (*p* = 0.34) (Table 1).

### 3.2. Etiology

The etiology of AIS in this study was intracranial vasculopathy in 10/28 (36%) patients (focal cerebral arteriopathy in eight cases, and cerebral vasculitis in two cases), cardiac disease in 6/28 (21%) patients (congenital heart disease in two cases, and cardiac surgery in four cases), and hematological disorders in 2/28 (7%) patients (acquired thrombophilia in both cases: nephrotic syndrome and antiphospholipid syndrome) (Table 1). Despite extensive research, 10/28 (36%) of the AIS cases remained idiopathic. Among the four AIS patients with recurrent stroke, one had intracerebral vasculitis, one had antiphospholipid syndrome, and two cases remained idiopathic.

For AHS, the most prevalent risk factor was vasculopathy in 9/16 (56%) patients (AVM in eight cases and aneurysm in one case); hematological causes were identified in 2/16 (13%) patients (one had thrombocytopenia due to hemophagocytic lymphohistiocytosis and the other had vitamin K deficiency); infection was the cause in 1/16 (6%) patients (she had acute meningoencephalitis); and 4/16 (25%) AHS cases remained idiopathic.

The risk factors for CVST were mild trauma in 1/5 (20%) patients, dehydration due to acute viral infection in 2/5 (40%) patients, and hematological disorders (homocystinuria and prothrombin mutation) in 2/5 (40%) patients.

### 3.3. Treatment

Pairwise comparison showed that children with AIS more often received conservative treatment compared to children with AHS. Acute treatment in the AIS subgroup was anticoagulation and/or anti-aggregation in 23/28 (82%) patients, thrombolysis in 2/28 (7.1%) and endovascular thrombectomy in 2/28 (7.1%) patients; one patient, 1/28 (3.6%), was operated on for suspected brain tumor (Table 1). In the AHS subgroup, acute treatment was operative in 9/16 (56%) (including two patients who died in the acute phase) and symptomatic in 7/16 (44%) patients. Two patients who had received only symptomatic treatment in the acute phase received operative treatment and endovascular treatment, respectively, later on. In the CVST subgroup, all patients were treated with anticoagulants.

### 3.4. Short-Term Outcome

Death in the acute stage of stroke occurred in 3/49 (6%) stroke patients, all in the AHS group (male, aged 1 year, 7 years, and 9 years). One patient with AIS died due to an underlying cardiac disease 2.5 months after stroke. Total mortality during the study period was 4/49 (8%): 1/28 (4%) in the AIS subgroup, 3/16 (19%) in the AHS subgroup, and 0/5 in the CVST subgroup (*p* = 0.14). Of the survivors, recurrent stroke occurred in 4/45 (8.9%) patients, all with AIS (Table 1) and in all cases in the first six months after initial stroke.

### 3.5. Long-Term Outcome

Post-stroke epilepsy occurred in 6/45 (13%) patients: 1/27 (4%) in the AIS subgroup, but as many as 5/13 (38%) in the AHS subgroup. There were no epilepsy cases in the CVST subgroup (0/5).

Long-term PSOM-based neurodevelopmental outcome was assessed in 41 patients at a median of 7.0 years after stroke, without differences between the subtypes (Table 2). The study revealed poor outcomes (PSOM ≥ 1) in 27/41 (66%) patients, with only 8/41 (20%) patients fully recovered (PSOM = 0) (Table 2). Outcomes were equally poor both in the AIS and AHS subgroups, with 17/24 (71%) and 9/13 (69%) patients, respectively (*p* > 0.99). Only one patient (1/4, 25%) had a poor outcome in the CVST subgroup.

Moderate–severe sensorimotor deficit (PSOM subscale score ≥ 1) was present in 21/41 patients (51%) (17, right, 4, left); moderate–severe language deficit in 6/41 patients (15%) (both in language production and language comprehension in four patients, in only language production in one patient and in only language comprehension in one patient); and moderate–severe cognitive/behavioral deficit was present in 5/41 patients (12%). In the sensorimotor subscale, a significant deficit (sensorimotor right-side and/or left-side, score ≥ 1) affecting wellbeing was present in 15/24 (63%) patients with AIS, in 6/13 (46%) patients with AHS, and in none of the patients with CVST (without differences between the different vascular groups).

There was no difference in the prevalence of any deficit (PSOM ≥ 0.5) in the PSOM subscales of right or left sensorimotor, language production, and language comprehension between the children with AIS, AHS or CVST (Table 3). However, significantly more children with AHS (10/13, 77%), compared to children with AIS (9/24, 38%), had problems in the cognition/behavior subscale (OR = 5.6 95%CI: 1.2–26, *p* = 0.022). The problems detected in the cognition/behavior subscale were related to cognition in 12/41 (29%) patients and behavior in 6/41 (15%) patients; three (7%) patients had combined problems with behavior and cognition.

Although patients with an initial Pediatric NIH Stroke Scale score ≥ 6 had worse outcomes according to the PSOM compared to patients with a Pediatric NIH Stroke Scale score < 6 (*p* = 0.0059), we were not able to identify any significant association for the AIS and AHS subgroups separately, nor were there differences in PSOM-based outcomes regarding gender, location of stroke, age at stroke or epilepsy (Table 4).

### 3.6. Combined Outcome

Combined outcomes in patients with known outcomes were available for 44 persons: for three patients who died of stroke and for 41 patients who participated in the outcome study and were evaluated for recurrent stroke, epilepsy, and the PSOM (Table 5). A combined poor outcome (epilepsy, PSOM ≥ 1, recurrent stroke, mortality) was seen in 75% of all patients with long-term outcome data, 75% of patients with AIS, 87.5% of patients with AHS, and 25% of patients with CVST. There was no significant difference between the AIS and AHS subgroups and between the AIS and CVST subgroups, but a statistically significant difference between the AHS and CVST subgroups was observed (*p* = 0.03, OR = 21 95%CI: 1.4–314).

## 4. Discussion

In our study, combined outcomes of childhood stroke (defined as death, total PSOM ≥ 1, recurrent stroke, and/or post-stroke epilepsy) were poor in 75% (33/44) of the patients with known outcomes, without differences between the patients with AIS and AHS. A similar Swedish study of outcomes after childhood stroke involving all stroke subtypes showed comparable results, with 65% of the patients having some neurological deficit [25].

According to our study, survivors of childhood stroke often experience impairments in multiple areas of development, which indicates the need for multidisciplinary teams for the rehabilitation and follow-up evaluation of these children.

None of the children in the AIS group died during the acute phase, which is concordant with our previous results [4]; still, reported mortality has been observed at 3.7% to 28% in AIS [34,35]. In our study, 19% of the children with AHS died in the acute phase, which is within a previously reported range (6 to 54%) [14].

Only children with AIS had recurrent stroke in our study (14%). Previous studies have reported a recurrence risk of 6% to 35% in AIS and 10% in AHS [34]. One explanation for the low stroke recurrence in our study is that since all participants were Caucasians, there were no cases of sickle cell disease or Moyamoya disease, which are the two preconditions for high stroke recurrence risk.

In our study, the prevalence of post-stroke epilepsy was 15%, while it was significantly higher in the AHS (38%) compared to the AIS subgroup (4%, *p* = 0.014). Earlier studies [36] have not reported this kind of difference, but those studies were retrospective and based on medical records. Some authors have reported the prevalence of post-stroke epilepsy in 27% of patients with childhood stroke (AIS and AHS combined) [36]. Several studies have found post-stroke epilepsy in 9–12% [18,37,38], and even in 41% [19] (when seizures after 48 h of stroke were included) of the AIS patients [18,19,37,38]. The potential cause for the very low number of epilepsy cases among the patients with AIS studied by us, compared to corresponding data from previous reports, remains unclear, even though the data from the medical records and the population-based database of prescriptions in our study was carefully checked to include all cases. In a subgroup of hemorrhagic stroke patients, epilepsy has been found in 15% of patients [12]; in patients with arteriovenous malformation rupture, epilepsy has been diagnosed in 16% of cases [39].

Although AHS accounts for nearly half of patients with childhood stroke, it has received far less attention than AIS in outcome studies. Previously, it has been shown that children with AHS have increased mortality, but lower morbidity compared to children with AIS [40,41]. Yet, our study did not confirm this finding.

### Neurodevelopmental Outcomes According to the PSOM

Neurocognitive outcomes according to the total PSOM were poor (moderate to severe damage) in 66% (27/41) of the survivors of childhood stroke, with only 20% of the patients fully recovered. This correlates with the finding of a recent study in childhood AIS patients, where 21.2% of the patients were discharged without deficit [35]. However, concerning neurodevelopmental outcomes according to total PSOM scores, the results did not differ between patients with AIS and AHS, with poor PSOM scores (PSOM ≥ 1) in 71% and 69% of the patients, respectively (*p* > 0.99).

In our study, patients with AHS had significantly more problems in the cognition/behavior PSOM subscale, compared to patients with AIS (77% vs. 38%, *p* = 0.022). Moderate–severe sensorimotor deficit was prevalent in 63% (15/24) of patients with AIS, in 46% (6/13) of the patients with AHS and in none of the CVST patients. Outcomes in the language and sensorimotor PSOM subscales were not different between the AIS and AHS subgroups. The occurrence of sensorimotor and cognitive problems is consistent with that reported by other authors; e.g., according to Blom et al., 48% of the patients have no physical deficit and 48% have no cognitive deficit [14,22].

As shown previously [20], more severe initial symptoms of stroke according to the PedNIHSS score are associated with worse long-term outcomes according to the PSOM, which was also found in our study.

There were no differences in PSOM-based neurodevelopmental outcomes, regarding gender, location of stroke (cortical or noncortical), age at stroke or development of epilepsy; this may be due to our relatively small study group.

Some authors have suggested that neonatal stroke and late childhood stroke (stroke between ≥6 and <16 years of age) are associated with better outcomes, whereas neonatal and early childhood AIS (stroke between 29 days and <6 years of age) lead to significantly worse outcomes in cognitive ability, processing speed, and verbal learning, irrespective of lesion size or location [42]. However, on the contrary, other studies have demonstrated that the group of early childhood AIS (stroke between ≥1 and <6 years of age) is characterized by significantly better outcomes than the infancy group (<1 year) and the late childhood group (≥6 years) [15], so the relationship between age at stroke and outcome is still unclear. Our study did not demonstrate an effect of age on stroke outcome according to the PSOM.

## 5. Limitations

One strength of this outcome study of childhood stroke is that despite the small study group, the data about long-term outcomes were available for 92% of the patients. Another strength is that 95% of the survivors had undergone follow-up MRI evaluation, which allowed to confirm the vascular subgroups of stroke and to assess the chronically affected brain areas.

However, there were only five patients in the CVST group, making the comparison between all vascular subgroups of stroke difficult. All patients diagnosed with CVST were in the younger subgroup (<6 years of age), which led us to believe that there may be problems diagnosing CVST in older children with unspecific symptoms. Young age has also been highlighted in other studies on CVST [21]. A second peak of CVST may occur in adolescence [43].

In our cohort, age at the time of stroke diagnosis is artificially lower, because the follow-up period lasted at least two years, so that older children had reached adulthood by the time of the outcome study. Hence, further studies including adults affected by childhood stroke are needed.

In assessing the patients’ outcomes, we did not consider their socioeconomic status or their parents’ education level. However, considering that medical treatment and rehabilitation are covered by universal healthcare and education (including higher education) is also universally provided by the state, we presume that the impact of the above aspects on the patients’ outcomes would not be significant.

It should be noted that actual neurodevelopmental outcomes may be better than that found in our study, as we believe that patients with good outcomes are less interested in participation; still, only four (4/45) patients declined to participate in the present study.

## 6. Conclusions

Combined outcomes of childhood stroke (epilepsy, PSOM ≥ 1, recurrent stroke, mortality) were poor in 75% (33/44) of the patients. Long-term outcomes in children with childhood stroke were equally poor in all vascular subtypes and only 20% of the children showed normal total PSOM scores. Only initial high Pediatric NIH Stroke Scale score was associated with poor neurodevelopmental outcomes according to the PSOM.

No differences in motor or language outcomes occurred between the different vascular subtypes of stroke, but mortality, rate of epilepsy and rate of poor cognitive outcomes were higher for children with AHS compared to children with AIS.

The findings of our study confirm the need for multidisciplinary follow-up and rehabilitation for all children with different vascular types of stroke.

## Figures and Tables

**Table 1:** Demographics and clinical characteristics of all patients with childhood stroke.

	Total N = 49	Arterial Ischemic Stroke (AIS) N = 28	Hemorrhagic Stroke (AHS) N = 16	Cerebral Venous Sinus Thrombosis (CVST) N = 5	*p* Value (Overall)	*p* Value (AIS vs. AHS)
Age at diagnosis, y, median (IQR)	4.0 (1.1–8.6)	4.1 (1.0–7.8)	7.9 (0.9–9.6)	2.4 (2.3–2.9)	0.34	0.53
Min, max	0.08, 14.6	0.13, 14.6	0.08, 11.3	0.17, 3.0		
Male gender, *n* (%)	27 (55)	16 (57)	8 (50)	3 (60)	0.91	0.65
Parenchymal lesion laterality, *n* (%)						
Unilateral	34 (69)	22 (79)	11 (69)	1 (20)	0.0001 ^a^	0.49 ^a^
Right	15/34 (44)	10/22 (46)	4/11 (36)	1/1 (100)	0.47 ^b^	0.72 ^b^
Left	19/34 (56)	12/22 (55)	7/11 (64)	0/1 (0)		
Bilateral	11 (22)	6 (21)	5 (31)	0 (0)	0.62 ^c^	0.49 ^c^
No intraparenchymal lesion	4 (8.2)	0 (0)	0 (0)	4 (80)		
Cortical lesion, *n* (%)	26 (53)	14 (50)	12 (75)	0 (0)	0.0084 *	0.10
Etiology						
Intracranial vasculopathy, *n* (%)	19 (39)	10 (36)	9 (56)	0 (0)	0.069	0.19
Hematological, *n* (%)	6 (12)	2 (7.1)	2 (13)	2 (40)	0.13	0.61
Cardiac, *n* (%)	6 (12)	6 (21)	0 (0)	0 (0)	0.093	0.072
Infections, *n* (%)	3 (6.1)	0 (0)	1 (6.3)	2 (40)	0.0092 **	0.36
Trauma, *n* (%)	1 (2.0)	0 (0)	0 (0)	1 (20)	0.10	N/A
Idiopathic, *n* (%)	14 (29)	10 (36)	4 (25)	0 (0)	0.34	0.46
Acute treatment, *n* (%)						
Conservative	35 (71)	23 (82)	7 (44)	5 (100)	0.0089 ***	0.0085
I/v thrombolysis	2 (4.1)	2 (7.1)	0 (0)	0 (0)		
Endovascular	2 (4.1)	2 (7.1)	0 (0)	0 (0)		
Operative	10 (20)	1 (3.6)	9 (56)	0 (0)		
Recurrent stroke, *n* (%) ****	4/45 (8.9)	4/27 (15)	0 (0)	0 (0)	0.41	0.28
Death, *n* (%)	4 (8.2)	1 (3.6)	3 (19)	0 (0)	0.14	0.13
Death in acute stage, *n* (%)	3 (6.1)	0 (0)	3 (19)	0 (0)	0.055	0.082

N, number of subjects; *n*, number of observations; IQR, interquartile range; min, minimum; max, maximum; OR, odds ratio; CI, confidence interval; N/A, not applicable; y, years. Comparison. ^a^ Between the groups with unilateral, bilateral and no intraparenchymal lesions; ^b^ between the groups with right-side and left-side lesions; ^c^ between the groups with unilateral and bilateral lesions, in AIS 6/28 (21%), in AHS 5/16 (31%), in CVST 0/1 (0%). * Pairwise comparison between the AIS and AHS groups, *p* = 0.10; between the AHS and CVST groups, OR = 31 95%CI: 1.4–671, *p* = 0.0062; between the AIS and CVST groups, *p* = 0.057. ** Pairwise comparison between the AIS and AHS groups, *p* = N/A; between the AHS and CVST groups, *p* = 0.13; between the AIS and CVST groups, OR = 41 95%CI: 1.6–1032, *p* = 0.019. *** Pairwise comparison between the AIS and AHS groups, OR = 5.9 95%CI: 1.5–24, *p* = 0.0085; between the AHS and CVST groups, *p* = 0.12; between the AIS and CVST groups, *p* = 0.57. **** Recurrent stroke assessed in 45 surviving patients.

**Table 2:** Demographics and clinical characteristics of the participants of the study with long-term neurological outcomes.

	Total N = 41	Arterial Ischemic Stroke (AIS) N = 24	Hemorrhagic Stroke (AHS) N = 13	Cerebral Venous Sinus Thrombosis (CVST) N = 4	*p* Value (Overall)	*p* Value (AIS vs. AHS)
Male gender, *n* (%)	21 (51)	14 (58)	5 (38)	2 (50)	0.53	0.25
Lesion laterality, *n* (%)						
Unilateral	28 (68)	18 (75)	9 (69)	1 (25)	0.002 ^a^	0.72 ^a^
Right	15 (37)	9/18 (50)	4/9 (44)	1/1 (100)	>0.99 ^b^	>0.99 ^b^
Left	14 (34)	9/18 (50)	5/9 (56)	0/1 (0)		
Bilateral	10 (24)	6 (25)	4 (31)	0 (0)	0.79 ^c^	0.72 ^c^
No intraparenchymal lesion	3 (7.3)	0 (0)	0 (0)	3 (75)		
Age at diagnosis, y, median (IQR)	4.3 (1.0–8.9)	4.6 (1.0–8.7)	8.5 (0.7–9.9)	2.3 (1.2–2.7)	0.35	0.86
Min, max	0.08, 14.6	0.13, 14.6	0.08, 11.3	0.17, 3.0		
Age at assessment, y, median (IQR)	14.8 (9.3–16.7)	14.5 (9.6–16.4)	16.1 (12.3–17.6)	7.3 (6.5–12.4)	0.14	0.23
Min, max	4.8, 18.8	4.8, 18.8	5.3, 18.8	6.3, 16.8		
Time from stroke, y, median (IQR)	7.0 (4.8–10.4)	6.8 (4.5–10.7)	7.5 (6.6–9.8)	5.0 (3.8–11.2)	0.56	0.61
Min, max	2.1, 17.4	2.1, 17.4	3.3, 17.3	3.3, 16.7		
Recurrent stroke, *n* (%)	4 (9.8)	4 (17)	0 (0)	0 (0)	0.39	0.28
Post-stroke epilepsy, *n* (%)	6 (15)	1 (4.2)	5 (38)	0 (0)	0.016 *	0.014
Post-stroke hydrocephalus	1 (2.4)	0 (0)	1 (7.7)	0 (0)	0.41	0.35
Cortical lesion, *n* (%)	22 (54)	12 (50)	10 (77)	0 (0)	0.0195 **	0.11
No chronic lesion, *n* (%)	4 (9.8)	0 (0)	0 (0)	4 (100)		
Total PSOM score, median (IQR)	1.0 (0.5–2.5)	1.3 (0.5–2.8)	1.5 (0.5–2.5)	0.3 (0.0–0.8)	0.13	0.86
Total PSOM, *n* (%)					0.28	>0.99
Normal (0)	8 (20)	4 (17)	2 (15)	2 (50)		
Mild (0.5)	6 (15)	3 (13)	2 (15)	1 (25)		
Moderate (1–1.5)	12 (29)	8 (33)	3 (23)	1 (25)		
Severe (≥2)	15 (37)	9 (38)	6 (46)	0 (0)		
Total PSOM, *n* (%)					0.26	>0.99
Good (<1)	14 (34)	7 (29)	4 (31)	3 (75)		
Poor (≥1)	27 (66)	17 (71)	9 (69)	1 (25)		
Total PSOM ≥ 1 and/or epilepsy, *n* (%)	29 (71)	17 (71)	11 (85)	1 (25)	0.091	0.45
PedNiHSS, ≥6, *n* (%)	16 (39)	10 (42)	6 (46)	0 (0)	0.29	0.79

N, number of subjects; *n*, number of observations; IQR, interquartile range; min, minimum; max, maximum; OR, odds ratio; CI, confidence interval; y, years. Comparison. ^a^ Between the groups with unilateral, bilateral and no intraparenchymal lesions; ^b^ between the groups with right-side and left-side lesions; ^c^ between the groups with unilateral and bilateral lesions, 6/24 (25%) in AIS, 4/13 (31%) in AHS, 0/1 (0%) in CVST. * Pairwise comparison between the AIS and AHS groups, OR = 14 95%CI: 1.2–708, *p* = 0.014; between the AHS and CVST groups, *p* = 0.26; between the AIS and CVST groups, *p* > 0.99. ** Pairwise comparison between the AIS and AHS groups, *p* = 0.11; between the AHS and CVST groups, OR = 27 95%CI: 1.1–638, *p* = 0.015; between the AIS and CVST groups, *p* = 0.11.

**Table 3:** Number of patients with problems in the different neurocognitive domains (subscale PSOM score ≥ 0.5).

PSOM Subscales	Total N = 41	Arterial Ischemic Stroke (AIS) N = 24	Hemorrhagic Stroke (AHS) N = 13	Cerebral Venous Sinus Thrombosis (CVST) N = 4	*p* Value (Overall)	*p* Value (AIS vs. AHS)
PSOM sensorimotor right, *n* (%)	23 (56)	15 (63)	7 (54)	1 (25)	0.41	0.61
PSOM sensorimotor left, *n* (%)	7 (17)	5 (21)	2 (15)	0 (0)	>0.99	>0.99
PSOM sensorimotor right or left, *n* (%)	29 (71)	20 (83)	8 (62)	1 (25)	0.033 *	0.23
PSOM language production, *n* (%)	12 (29)	8 (33)	4 (31)	0 (0)	0.53	>0.99
PSOM language comprehension, *n* (%)	8 (20)	5 (21)	3 (23)	0 (0)	0.86	>0.99
PSOM language production, comprehension, *n* (%)	12 (29)	8 (33)	4 (31)	0 (0)	0.53	>0.99
PSOM cognition/behavior, *n* (%)	21 (51)	9 (38)	10 (77)	2 (50)	0.086 **	0.022

N, number of subjects; *n*, number of observations. * Pairwise comparison between the AIS and AHS groups, *p* = 0.23; between the AHS and CVST groups, *p* = 0.29; between the AIS and CVST groups, OR = 15 95%CI: 1.2–184, *p* = 0.038. ** Pairwise comparison between the AIS and AHS groups, OR = 5.6 95%CI: 1.2–26, *p* = 0.022; between the AHS and CVST groups, *p* = 0.54; between the AIS and CVST groups, *p* > 0.99. Note. The values are expressed as absolute counts and percentages. N, number of subjects; *n*, number of observations.

**Table 4:** Factors affecting total PSOM.

	Total PSOM				*p* Value
	*n*	Median (IQR)	Min	Max	
Gender					0.17
Male	21	1.5 (1–2.5)	0	6	
Female	20	1 (0.5–1.8)	0	5	
Lesion laterality					
Unilateral	28	1 (0.5–2.5)	0	3.5	0.094 ^a^
Right	14	1.5 (0.5–2.5)	0	3	0.76 ^b^
Left	14	1 (0.5–2)	0	3.5	
Bilateral	10	1.5 (0–3.5)	0	6	0.69 ^c^
No intraparenchymal lesion	3	0 (0–0.5)	0	0.5	
Lesion location					0.60
Cortical	22	1.5 (0.5–3)	0	6	
Noncortical	16	1 (1–2)	0	3.5	
Epilepsy					0.35
No	35	1 (0.5–2.5)	0	5	
Yes	6	1.8 (0.5–3)	0.5	6	
Age at stroke onset					0.99
<6.0 years	23	1 (0.5–2.5)	0	6	
≥6.0 years	18	1 (0.5–2.5)	0	3.5	
Treatment					0.15
Conservative	31	1 (0.5–2.5)	0	5	
Invasive	10	1.8 (1–3)	0	6	
PedNiHSS					0.0059
≥6	16	2.0 (1.0–3.3)	0	6	
<6	25	0.5 (0–1.5)	0	3.5	

*n*, number of observations; IQR, interquartile range; min, minimum; max, maximum. Comparison. ^a^ Between the groups with unilateral, bilateral and no intraparenchymal lesions; ^b^ between the groups with right-side and left-side lesions; ^c^ between the groups with unilateral and bilateral lesions.

**Table 5:** Components of combined poor outcome.

	Total N = 44	Arterial Ischemic Stroke (AIS) N = 24	Hemorrhagic Stroke (AHS) N = 16	Cerebral Venous Sinus Thrombosis (CVST) N = 4	*p* Value (Overall)	*p* Value (AIS vs. AHS)
Recurrent stroke, *n* (%)	4/41 (9.8)	4 (17)	0/13 (0)	0 (0)		
Post-stroke epilepsy, *n* (%)	6/41 (15)	1 (4.2)	5/13 (38)	0 (0)		
Total PSOM ≥ 1	27/41 (66)	17 (71)	9/13 (69)	1 (25)		
Death in acute stage, *n* (%)	3 (6.9)	0 (0)	3 (19)	0 (0)		
Combined poor outcome (epilepsy, PSOM ≥ 1, recurrent stroke, mortality), *n* (%)	33 (75)	18 (75)	14 (87.5)	1 (25)	0.035 *	0.44

N, number of subjects; *n*, number of observations; IQR, interquartile range; min, minimum; max, maximum; OR, odds ratio; CI, confidence interval; y, years. * Pairwise comparison between the AIS and AHS groups, *p* = 0.44; between the AHS and CVST groups, OR = 21 95%CI: 1.4–314, *p* = 0.032; between the AIS and CVST groups, *p* = 0.084.

## Data Availability

The original contributions presented in this study are included in the article. Further inquiries can be directed to the corresponding author.
